# Drug-induced liver injury associated with dacomitinib: A case report

**DOI:** 10.3389/fonc.2022.979462

**Published:** 2022-09-14

**Authors:** Xuanxuan Wang, Anqi Huang, Yun Lu, Suyu Gao, Wen Hu, Hong Cheng

**Affiliations:** Department of Pharmacy, Zhongnan Hospital of Wuhan University, Wuhan, China

**Keywords:** dacomitinib, drug-induced liver injury, non-small cell lung cancer, Roussel Uclaf Causality Assessment Method, case report

## Abstract

Dacomitinib, the second-generation epidermal growth factor receptor tyrosine kinase inhibitor (EGFR-TKI), has been used as a first-line treatment in non-small cell lung cancer (NSCLC) patients harboring EGFR mutation. In this case, we report a patient with drug-induced liver injury (DILI) associated with the use of dacomitinib. A 59-year-old man with stage IV NSCLC was prescribed with dacomitinib; 37 days after dacomitinib administration, he was admitted to our hospital because of jaundice. Laboratory examinations revealed elevated serum levels of liver enzymes and bilirubin. Following the immediate discontinuation of dacomitinib, liver enzymes decreased but bilirubin continued to rise. Total bilirubin reached the peak (18-fold) on day 26 after dacomitinib termination and normalized on day 146 after dacomitinib discontinuation. A “probable” cause of DILI by dacomitinib was determined based on the Roussel Uclaf Causality Assessment Method. The severity of DILI was assessed as acute liver failure. To our knowledge, this is the first case of DILI caused by dacomitinib monotherapy in a real-world setting. Clinicians should pay particular attention to the possibility of DILI during dacomitinib treatment.

## Introduction

Lung cancer remains the leading cause of cancer incidence and mortality worldwide. Non-small cell lung cancer (NSCLC) accounts for 80%–90% of lung cancers (1). In addition, epidermal growth factor receptor (EGFR) gene mutations occur in 10%–44% of lung adenocarcinomas, a common type of NSCLC (2).

To date, epidermal growth factor receptor tyrosine kinase inhibitors (EGFR-TKIs) have been playing irreplaceable roles in the treatment of NSCLC patients harboring EGFR mutation (3, 4). Dacomitinib, the second-generation EGFR-TKI, is an oral, small-molecule irreversible inhibitor of the EGFR family. Available in 2018, dacomitinib is approved for use in locally advanced or metastatic NSCLC patients harboring EGFR exon 19 deletion or exon 21 L858R substitution by the FDA. There have been no reports of drug-induced liver injury (DILI) caused by dacomitinib monotherapy in real-world environments to date.

In this study, we describe the first case of DILI associated with dacomitinib monotherapy in a real-world setting, marked by an 18-fold increase in bilirubin levels.

## Case presentation

**Figure 1:**
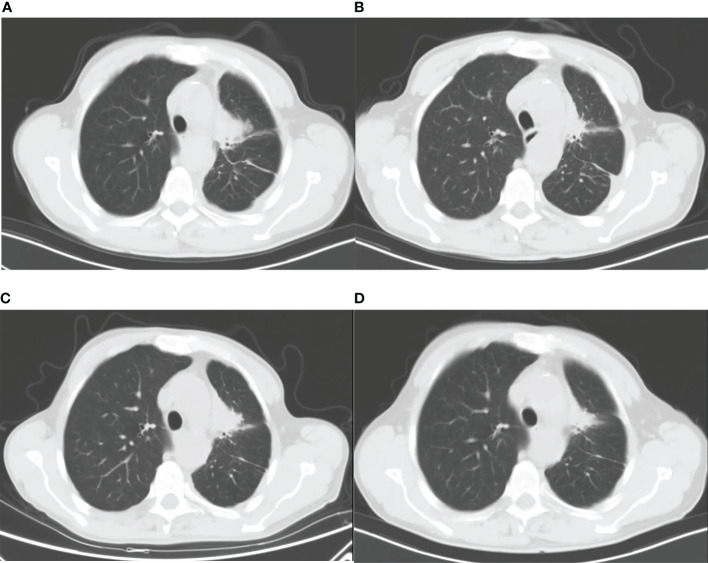
Lung CT images: **(A)** before dacomitinib administration; **(B)** 37 days after dacomitinib administration; **(C)** 50 days after dacomitinib termination; and **(D)** 95 days after afatinib administration.

On 1 December 2021, a previously healthy 59-year-old man was diagnosed with stage IV lung adenocarcinoma harboring L858R mutation. He denied a history of hypertension, diabetes, coronary heart disease, liver diseases, or infectious diseases such as tuberculosis. A 40 × 33 × 46 mm mass in the paramediastinal of the upper lobe of the left lung is shown in **Figure 1A**. The patient had no family history of cancer and other diseases and no history of drugs or food allergies. He had a long history of smoking (15–25 cigarettes/day) and drinking (150–200 ml/day) for more than 30 years and quit smoking and drinking after his diagnosis of lung cancer. At the time, he had normal liver function with laboratory indicators as follows: alanine aminotransferase (ALT) at 10 U/L (normal range, 9–50 U/L), aspartate aminotransferase (AST) at 14 U/L (normal range, 15–40 U/L), alkaline phosphatase (ALP) at 73 U/L (normal range, 30–120 U/L), γ-glutamyltransferase (GGT) at 14 U/L (normal value, 8–57 U/L), total bilirubin (TBil) at 9.8 µmol/L (normal value, 5–21 µmol/L), direct bilirubin (DBil) at 2.1 µmol/L (normal value, 0–7 µmol/L), indirect bilirubin (IBil) at 7.7 µmol/L (normal value, 1.5–18 µmol/L), prothrombin time activity (PTA) at 84% (normal value, 80–130%), and international normalized ratio (INR) at 1.11 (normal value, 0.85–1.15). Five days later, he received one dose of cisplatin (40 mg, intrapleural perfusion) and initiated targeted therapy with dacomitinib (45 mg daily, oral). He was then discharged on day 16.

On the 37th day of dacomitinib administration, the patient was readmitted with jaundice. As shown in **Figure 1B**, the tumor size was significantly reduced compared with pre-treatment (**Figure 1A**). He had no other symptoms except for obvious yellowing of the skin and sclera. Laboratory examinations indicated liver injury: ALT at 324 U/L, AST at 176 U/L, ALP at 739 U/L, GGT at 523 U/L, TBil at 160.2 µmol/L, DBil at 95.8 µmol/L, IBil at 64.4 µmol/L, and bilirubin detected in urine. The patient tested negative for hepatitis A virus antibody (anti-HAV-IgM), hepatitis B virus antigen and DNA (HBsAg, HBV DNA), hepatitis C virus antigen and RNA (HCV cAg, HCV RNA), hepatitis E virus antibody (anti-HEV-IgM/IgG), and autoantibodies (including antinuclear antibodies, smooth muscle antibodies, antibodies to the liver–kidney microsome type 1, antimitochondrial antibodies, antibodies to liver cytosol type 1, and antibodies to soluble liver antigen). With 4.27 ng/ml of alpha-fetoprotein (AFP) in the normal range (0.89–8.78 ng/ml), abdominal ultrasound and computed tomography (CT), liver magnetic resonance imaging (MRI), and magnetic resonance cholangiopancreatography (MRCP) show no abnormality.

The Naranjo score for dacomitinib-induced liver injury was 5, indicating probable adverse drug reactions. It was determined that dacomitinib was a “probable” cause of DILI based on a Roussel Uclaf Causality Assessment Method (RUCAM) score of 8 (5). In addition, the cholestatic type of DILI caused by dacomitinib was diagnosed according to DILI guidelines (6, 7). A liver biopsy was not performed due to the poor general condition of the patient.

**Figure 2:**
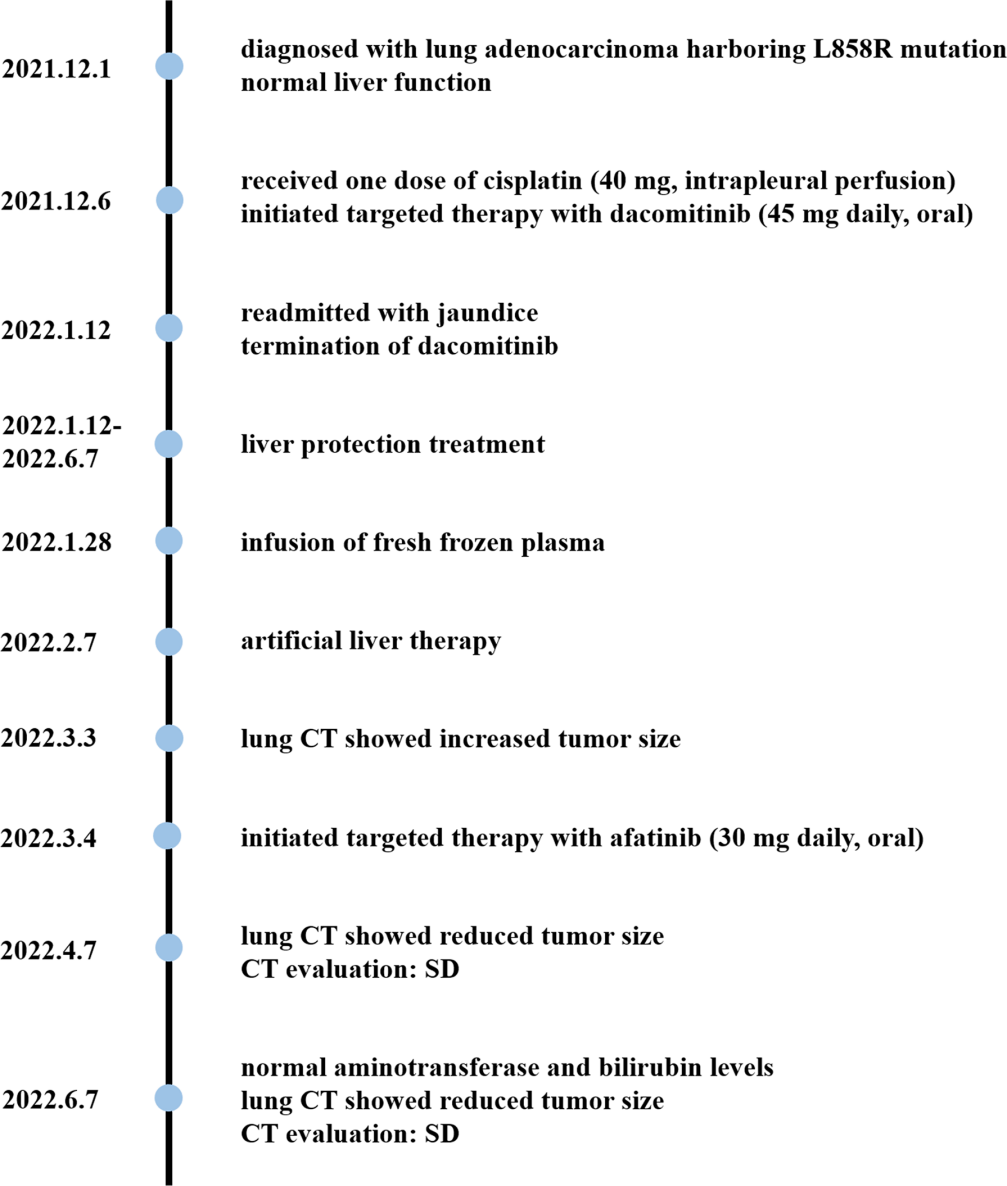
Timeline of interventions and outcomes.

The timeline is shown in **Figure 2**. Dacomitinib was discontinued on the first day of readmission. The patient then received drug and non-drug therapy, including magnesium isoglycyrrhizinate at 100 mg daily, S-adenosylmethionine at 1 g daily, and ursodeoxycholic acid at 250 mg three times daily throughout the hospital stay; *N*-acetylcysteine at 8 g daily between the 12th and 17th hospital days and infusion of plasma on the 16th hospital day because of a sudden deterioration of coagulation function; and once artificial liver therapy on the 26th hospital day due to high levels of bilirubin. The patient was discharged on the 29th hospital day (29 days after dacomitinib termination) and continued ursodeoxycholic acid treatment outside the hospital. He was followed up for 4 months.

**Figure 3:**
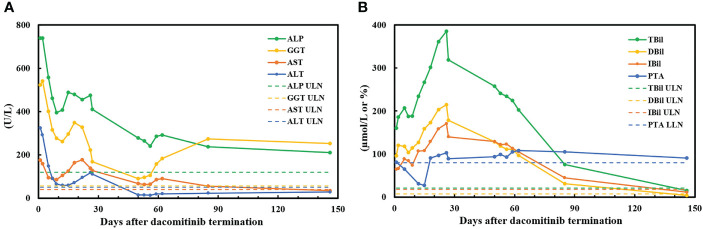
Serum levels of laboratory parameters in the patient. **(A)** Alanine aminotransferase (ALT), aspartate aminotransferase (AST), alkaline phosphatase (ALP), and γ-glutamyltransferase (GGT); **(B)** total bilirubin (TBil), direct bilirubin (DBil), indirect bilirubin (IBil), and prothrombin time activity (PTA). ULN, upper limit of normal; LLN, lower limit of normal.

During hospitalization, the patient remained sane without signs of hepatic encephalopathy. However, he developed a change in taste, poor appetite, abdominal distension, generalized itching, aggravated yellowing of the skin and sclera, yellow urine, and white stool. The serum levels of the laboratory parameters in the patient are shown in **Figure 3**. After the discontinuation of dacomitinib, the liver enzyme (ALT, AST, ALP, GGT) levels decreased obviously. However, bilirubin (TBil, DBil, IBil) levels showed an upward trend, reaching the highest (18-fold) level on day 26 after dacomitinib termination. It subsequently decreased partly after artificial liver therapy, followed by a gradual downward trend, and finally normalized on day 146 after dacomitinib discontinuation. Coagulation function (PTA) was relatively stable initially, decreased significantly (PTA<30%) on day 15 after dacomitinib termination, recovered, and remained in the normal range after an infusion of fresh frozen plasma (350 ml) on day 16 after dacomitinib termination. The severity of DILI was assessed as acute liver failure (ALF) based on DILI guidelines (6, 7).

Fifty days after dacomitinib termination, a lung CT image showed an increased tumor size (**Figure 1C**), and the patient received afatinib 30 mg daily the next day. 34 and 95 days after afatinib administration, lung CT showed a reduced tumor size without tumor progression and metastasis (**Figure 1D**). The patient achieved stable disease (SD) based on imaging assessment and continued to receive afatinib treatment. The patient showed agreement on the risk, medication, and treatment plan with the clinician throughout the entire treatment.

## Discussion

DILI is a diagnosis of exclusion and requires careful assessment. In this case, as mentioned above, the patient reported no family history. He remained sane and developed a change in taste, poor appetite, abdominal distension, generalized itching, jaundice with elevated liver enzymes and bilirubin levels during hospitalization. Liver imaging showed no abnormality. The patient presented no symptoms of fever or lymphadenopathy, and extrahepatic manifestations of HEV infection, especially neurological injury, were not observed during hospitalization (8). Laboratory parameters for viral hepatitis and autoantibodies were all negative. Significantly elevated ALP levels were not consistent with a typical pattern of autoimmune liver diseases. These lead to an unsupported diagnosis of viral hepatitis and autoimmune hepatitis (9). Although the patient had a long history of drinking, abnormal indicators of alcoholic hepatitis, such as AST/ALT >1.5, were not observed. Steatosis and cirrhosis were also not found in the liver imaging. The diagnosis of alcohol-associated liver diseases and nonalcoholic steatohepatitis was then excluded (10). The patient presented no Kayser–Fleischer rings and neuropsychiatric disturbances, the most common presentations of Wilson disease, and reported no family history, so Wilson disease could be excluded (11). The dacomitinib-induced liver injury was finally established based on the Naranjo score of 5 and the RUCAM score of 8. However, Epstein–Barr virus, cytomegalovirus, and further examinations for HEV were not tested after the clinician empirically established the high possibility of DILI, which also made the RUCAM and Naranjo scores not higher.

Several studies have demonstrated the efficacy of dacomitinib in EGRF-mutated NSCLC patients. In the ARCHER 1050 study (NCT01774721), compared with the first-generation EGRF-TKI gefitinib, dacomitinib showed significant improvement in progression-free survival and overall survival (12, 13). The better efficacy in Asians and patients with EGFR exon 21 L858R mutation was also proven (14, 15).

However, growing evidence shown the toxicities of dacomitinib may limit its use in clinical practice (16). Adverse events (including laboratory abnormalities) are assessed and graded using the National Cancer Institute Common Terminology Criteria for Adverse Events (CTCAE) version 4.0. Adverse events (of any cause) occurred in almost all patients given dacomitinib. The most common adverse events (of any grade) of dacomitinib are diarrhea, paronychia, dermatitis acneiform, and stomatitis. Among them, the most commonly reported adverse events in grades 3–5 are dermatitis acneiform and diarrhea (12). Treatment-related serious adverse events may lead to permanent discontinuation. In patients with dacomitinib, they are gastrointestinal disorders and skin and subcutaneous disorders.

Liver injury is not a major adverse event of dacomitinib. In the ARCHER 1050 study, when taking adverse events of any cause into consideration, 19% of patients experienced grades 1–2 adverse events with elevated liver enzymes (ALT, AST), and only two patients (1%) developed a grade 3 adverse event of ALT elevation. In terms of treatment-related serious adverse events, no patients suffered liver injury such as elevated liver enzymes and bilirubin (12). The incidence of hepatotoxicity associated with dacomitinib was less frequent than with the first-generation EGFR-TKI gefitinib (12, 17). Overall, the frequency of severe aminotransferase abnormalities and DILI with dacomitinib showed a lower risk. Hepatotoxic events potentially related to dacomitinib reported in clinical trials were mostly transient increased aminotransferase levels (18). A network meta-analysis of hepatotoxicity with EGFR-TKIs revealed an uncertain association between dacomitinib and the risk of liver enzyme elevation in patients diagnosed with NSCLC (19). After consulting the literature, only one Chinese case report was involved, which was about DILI marked by elevated liver enzymes owing to the combined use of dacomitinib and metoprolol (20).

Dacomitinib is mainly metabolized by the liver through oxidative and conjugative metabolism (21). In this study, dacomitinib-induced liver injury was a near-certainty diagnosis after assessing with RUCAM, Naranjo’s adverse drug reaction probability scale, and analyzing clinical, laboratory, and imaging features. The DILI case was mainly marked as an 18-fold increase in bilirubin levels accompanied by an increase in liver enzymes, which was defined as a grade 3 adverse event. Since severe DILI is a very rare complication of dacomitinib therapy, the monitoring of liver function received little attention. The occurrence of liver injury was not detected until the patient was readmitted because of jaundice a month later. Therefore, the surveillance of liver function during treatment is necessary for the early detection of hepatotoxicity.

Actually, dacomitinib-associated adverse events are manageable with treatment interruption, dose reduction, and/or adjuvant drug therapy (12). In the ARCHER 1050 study, dose reduction did not reduce the efficacy of dacomitinib despite a prolonged overall treatment time. Also, the incidence and severity of adverse events were effectively decreased (13). This suggests that dosage reduction may be the best solution for relieving adverse events induced by dacomitinib (17). Nearly two-thirds of patients taking dacomitinib required at least one dose modification (22). However, in this study, dacomitinib-induced grade 3 hepatotoxicity and disease progression revealed by tumor imaging assessments (CT), led to the permanent withdrawal of dacomitinib and switch to afatinib. In some cases, patients who have suffered hepatotoxicity due to EGFR-TKIs were successfully switched from one EGFR-TKI to another (23, 24).

It is worth mentioning how to rule out the possibility of cisplatin-induced liver injury. First, cisplatin is metabolized by the kidneys, generally showing nephrotoxic, bone marrow toxicity, and rarely hepatotoxicity. Secondly, patients received cisplatin *via* local chemotherapy of intrapleural perfusion, reducing the influence on the liver in the abdominal. Finally, adverse reactions to cisplatin are commonly immediate. As cholestatic-type DILI, the liver injury occurred 37 days after the withdrawal of cisplatin, which was assessed as “no correlation” according to the RUCAM scale.

Furthermore, possible increased toxicity due to drug interactions is of concern. Dacomitinib is mainly metabolized in liver microsomes by CYP2D6 with a major metabolite as *O*-desmethyl dacomitinib (25). For patients taking dacomitinib, concomitant use of CYP2D6 inhibitors (such as paroxetine) or CYP2D6 substrates (such as trazodone) should be avoided to prevent an increased risk of drug toxicity (26, 27). Meanwhile, dacomitinib is a substrate/an inhibitor of adenosine triphosphate (ATP)-binding cassette (ABC) transporters including P-glycoprotein (P-gp) and breast cancer resistance protein (BCRP) on the cell membrane surface, which are related to tumor multidrug resistance (MDR) (28). In addition, concomitant use of acid-suppressing drugs possibly reduces dacomitinib exposure through reducing the solubility of dacomitinib. In this case, from receiving chemotherapy for the first time (including dacomitinib and one dose of cisplatin by intrapleural infusion) and discharging with normal liver function to readmitting with jaundice, the patient only took dacomitinib outside the hospital because of the absence of other underlying diseases. Cisplatin is metabolized *via* nonenzymatic mechanisms, not involving the CYP2D6 pathway. Therefore, the use of cisplatin is less likely to cause increased drug toxicity. Dacomitinib, on the other hand, has been shown to improve cisplatin chemosensitivity and generate synergistic antitumor effects by inhibiting the function of P-gp and BCRP (29). Cisplatin can increase the expression of ABC transporters, including P-gp and BCRP. The overexpression of P-gp and BCRP is one of the mechanisms of cisplatin resistance. Downregulating the expression of P-gp and BCRP may be a promising strategy to overcome cisplatin resistance and enhance antitumor activity (30).

There are still some limitations to this study. The lack of pathological examination and serological tests for Epstein–Barr virus, cytomegalovirus, and reverified HEV infection makes the diagnosis of DILI still challenging. The association between drug metabolism genotype and liver injury is not fully explored owing to the lack of genetic testing, and the mechanism of dacomitinib-induced liver injury remains unclear.

## Conclusions

To our knowledge, this is the first case report of DILI caused by dacomitinib monotherapy in a real-world setting. Clinicians should pay particular attention to the possibility of DILI during dacomitinib treatment.

## Data availability statement

The original contributions presented in the study are included in the article/supplementary material. Further inquiries can be directed to the corresponding author.

## Ethics statement

Written informed consent was obtained from the individual(s) for the publication of any potentially identifiable images or data included in this article.

## Author contributions

XW conceived of the design and wrote the manuscript. AH, YL, SG, and WH revised the manuscript. HC reviewed the manuscript and assumed responsibility for data completeness and accuracy. All authors contributed to the article and approved the submitted version.

## Conflict of interest

The authors declare that the research was conducted in the absence of any commercial or financial relationships that could be construed as a potential conflict of interest.

## Publisher’s note

All claims expressed in this article are solely those of the authors and do not necessarily represent those of their affiliated organizations, or those of the publisher, the editors and the reviewers. Any product that may be evaluated in this article, or claim that may be made by its manufacturer, is not guaranteed or endorsed by the publisher.
